# A delayed spontaneous second-trimester tubo-abdominal pregnancy diagnosed and managed by laparotomy in a “self-identified” infertile woman, a case report and literature review

**DOI:** 10.1186/s12884-023-05793-1

**Published:** 2023-07-13

**Authors:** Yanfang Zhang, Mengying Li, Xiaomei Liu, Wen Yang, Qingyun Dong, Dan Wang, Jinghua Wang, Wenyan Tian, Xueru Song

**Affiliations:** 1 Tianjin Key Laboratory of Female Reproductive Health and Eugenics, Tianjin, China; 2 Laboratory of Epidemiology, Tianjin Neurological Institute & Department of Neurology, Tianjin Medical University General Hospital, Tianjin, China; 3 Department of Obstetrics and Gynecology, Tianjin Medical University General Hospital, 154 Anshan Road, He Ping District, Tianjin, 300052 China

**Keywords:** In vitro fertilization history, Abdominal pregnancy, Tubal-abdominal pregnancy, Second-trimester, Polycystic ovary syndrome

## Abstract

**Background:**

Abdominal pregnancy, a rare form of ectopic pregnancy, is associated with high morbidity and adverse consequences for future fertility. Early recognition and management reduce mortality and allow minimal invasive and conservative treatment. In modern medicine, primitive prevention to unexpected fatal pregnancies is crucial.

**Case presentation:**

A divorced 33-year-old “self-identified” infertile polycystic ovary woman diagnosed as repeated implantation failure in previous in vitro fertilization with her ex-husband ever presented in surgery department with a history of 15-day abdominal pain, nausea, and vomiting and 3-h worsening abdominal pain. The serum beta-human chorionic gonadotropin value was more than 10,000 m-international units per milliliter. Sonogram findings were significant for the absence of intrauterine gestation; a placenta and well-formed living fetus of second-trimester gestation were seen in the abdomen, accompanied by hemoperitoneum. A unique spontaneously second-trimester tubo-abdominal pregnancy was confirmed in emergent laparotomy by gynecologists, she received a removing of the living fetus, a right total salpingectomy, resection of partial omentum and blood transfusion. The patient recovered uneventfully and her serum beta-human chorionic gonadotropin returned to normal range on the 30th postoperative day, till now, she has weak fertility awareness because of her catastrophic experiences in the unexpected abdominal pregnancy.

**Conclusions:**

This case highlights woman with a previous in vitro fertilization history may be in is a high risk to be delayed or missed in diagnosis in an intended ectopic pregnancy due to a fixed belief in infertility. Educational interventions and contraceptive care should be provided by fertility and healthcare practitioner. The possibility of abdominal pregnancy must always be suspected and dealt with promptly and appropriately by the astute clinician.

**Supplementary Information:**

The online version contains supplementary material available at 10.1186/s12884-023-05793-1.

## Background

In more than 95% of cases, ectopic pregnancies (EPs) occur in the fallopian tube [1]. Rare cases are found as cervical, ovarian, cesarean scar, interstitial, cornual, or abdominal ectopic pregnancies (APs) [2, 3]. Often due to delayed or missed diagnosis, these uncommon forms of EPs have been associated with significant morbidity and mortality. Take AP for example, it represents only 1% of EPs but which has a maternal mortality rate eight times greater than tubal pregnancies [4]. APs are classified as either primary or secondary. Early APs (< 20 weeks) and advanced ones (≥ 20 weeks) are categorized also.

Diagnosis and treatment of APs are challenging in delayed cases after the first trimester, especially in advanced APs, the ones generally presented with grievous consequences due to the nonspecific signs and symptoms. Diagnostic laparotomy is traditionally reserved in case hemodynamically unstable. Dilemma lies on the management of abnormal implantation of placenta, which can cause severe maternal postpartum hemorrhage, organ injury, infection, coagulopathy, which could lead to death in severe cases [5–7].

Advances in ultrasound technology and quantitative measurement of the beta subunit of human chorionic gonadotropin (beta-hCG) over recent years have led to an increase in the early diagnosis of tubal EPs and APs. As a consequence, it is possible that the clinical presentation of APs, a life-threatening disease necessitating emergency diagnostic surgery changes into a more benign and stable condition. A trend in the later case reports indicates early APs with or without intraperitoneal hemorrhage localized in the pelvic cul-de-sac, broad ligament, bowel, or pelvic sidewall, liver, spleen and so on can all be treated via laparoscopy successfully [8–13], there are reports of early APs being treated successfully with minimal invasive techniques and the exploration of medical and conservative treatments [14–16]. Feasibility of early recognition allows more individual interventions to decrease mortality and increase fertility preservation.

In modern medicine, a “self-identified” infertile polycystic ovarian syndrome (PCOS) woman having previous in vitro fertilization (IVF) procedure history, was emergently admitted at second trimester gestation with a massive hemoperitoneum. Successfully treated in emergency but with worse fertility prognosis. Our case is unique in highlights a special attention should be given to the woman following discontinuation of previous IVF treatment, due to the lack of information on the probability of spontaneous pregnancy and wrong perception of not pursing assisted conception is an active contraceptive choice, they should be high-risk population of being delayed or misdiagnosed in naturally conceived EPs or APs.

## Case presentation

A 33-year-old nulliparous divorced PCOS woman was admitted to the surgical emergency department complaining of 15 days of abdominal pain, nausea, and vomiting that had worsened within the 3 h prior to presentation. An appointment for a gastroenteroscopy was made a week ago complaining of digestive symptoms. The patient was admitted to the gynecological department for multidisciplinary consultation.

The woman reported that her last menstrual period occurred 37 days ago lasting 8 days long. Her next most recent menstrual period occurred 3 months more ago, presenting as a small amount of brown vaginal bleeding over the course of 1 month. She was diagnosed as PCOS 8 years ago. Her menstrual history had been significant for bleeding that lasted for 8–20 days, with irregular periods, 30–90 days apart.

She revealed a 4-year history of primary infertility with her ex-husband for which she had undergone IVF and failed to obtain a clinical pregnancy after three times of freeze–thaw embryo transfers in total with six good quality embryos on day 3, which could be diagnosed as repeated implantation failure (RIF) [17].

The patient denied the possibility of pregnancy. As a “self-identified” infertile woman, she divorced with her ex-husband one year ago, and started intercourse with her new boyfriend six month ago without contraception.

Her pulse rate was125 bpm/min and blood pressure indicated 99/50 mmHg. She was pale, lethargic, and demonstrated abdominal tenderness.

**Fig. 1:**
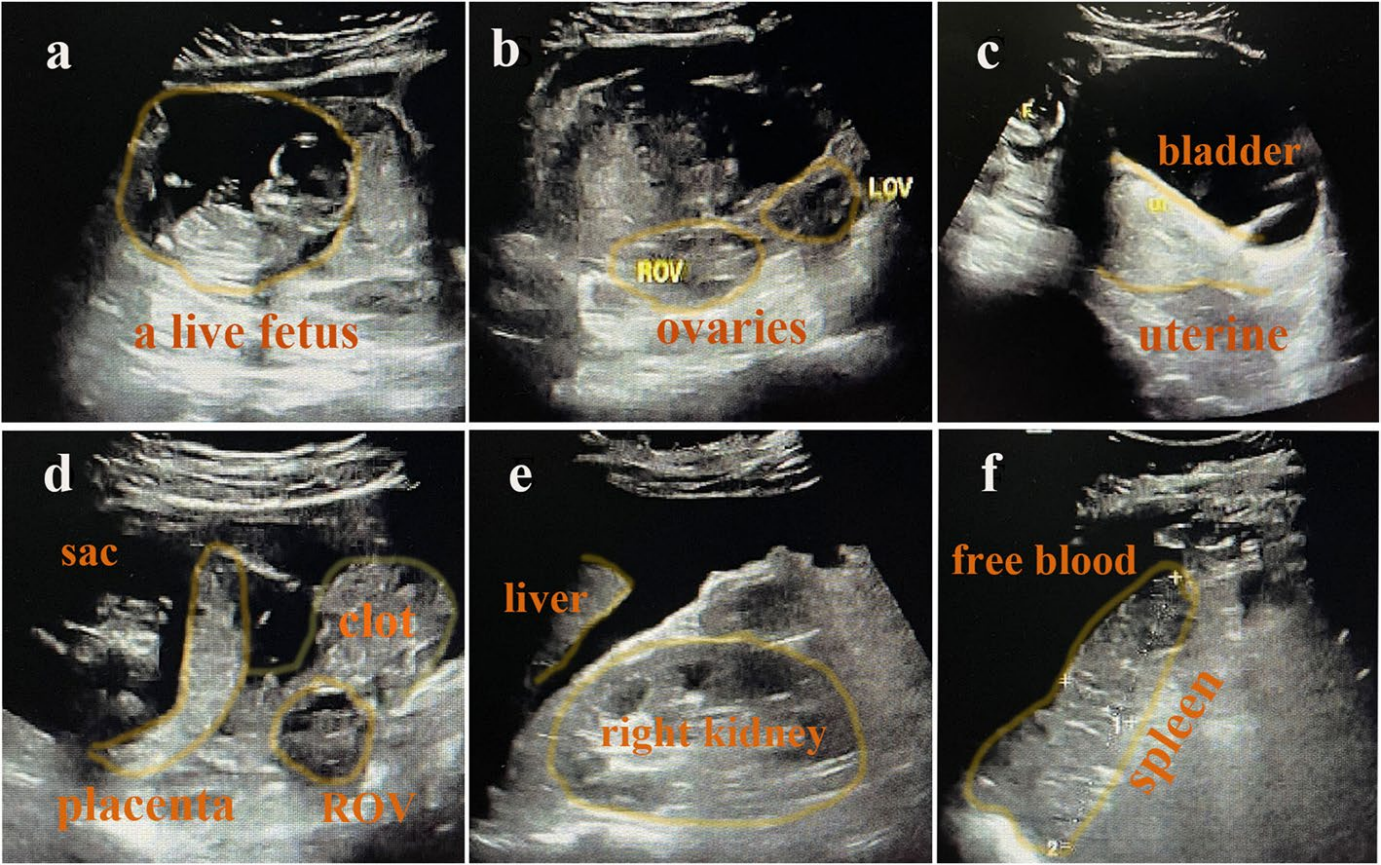
Transabdominal ultrasound images. ROV:right ovary, LOV:left ovary

A rapid urine pregnancy test was administered, and the positive result was unexpected. Other significant laboratory values included those for hemoglobin (59 g/L) and beta-hCG was more than 10,000 m-international units per milliliter. Sonogram findings were significant for the absence of intrauterine gestation; a placenta and well-formed living fetus were seen in the abdomen, to the upper right of the uterus, accompanied by moderate free fluid within the abdomen (Fig. 1). The fetus had a crown-rump length of 6.1 cm, corresponding to 12 weeks and 4 days of gestation, the fetal heart rate was176 bpm. A spontaneous conceived AP was ensured.

**Fig. 2:**
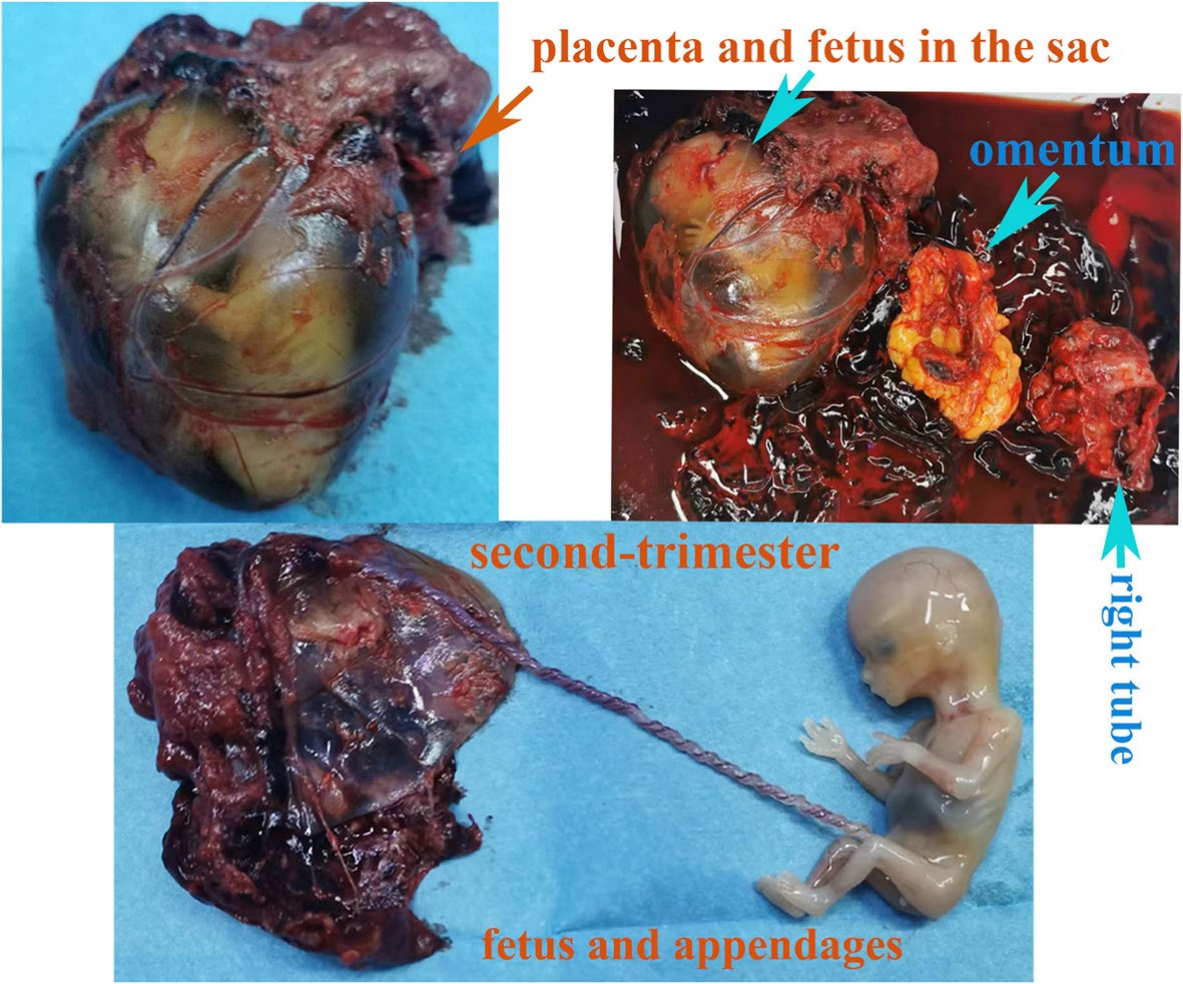
Surgical specimens

The patient underwent an emergency laparotomy (Fig. 2). The hemoperitoneum (3000 mL) was visualized and evacuated. Mostly dissected placental tissues and a fetus in an intact amniotic sac (9 cm × 6 cm × 5 cm) were seen in the upper part of the abdominal cavity. Part of the placenta had implanted into the omentum. The ampulla of the right fallopian tube was obviously thickened and congested, with a 4 cm rupture and some residual placental tissue along the ruptured tube. Persistent oozing of blood was also observed. Except for the PCOS appearance of both ovaries, slight inflammation had been seen on their surface. The uterus and left fallopian tube were normal. There was no obvious evidence of endometriosis. Moreover, there was no evidence of damage to the other organs in the pelvic and abdominal cavities. A surgery performed successfully with removal of abdominal gestation tissue, resection of partial omentum and right salpingectomy. Intraoperative infusions of 910 mL of autogenous blood and 2 units of concentrated red blood cells were required.

Five days after laparotomy, the patient’s serum hCG level had declined to 696 IU/L and was normal 1 month after the operation.

**Fig. 3:**
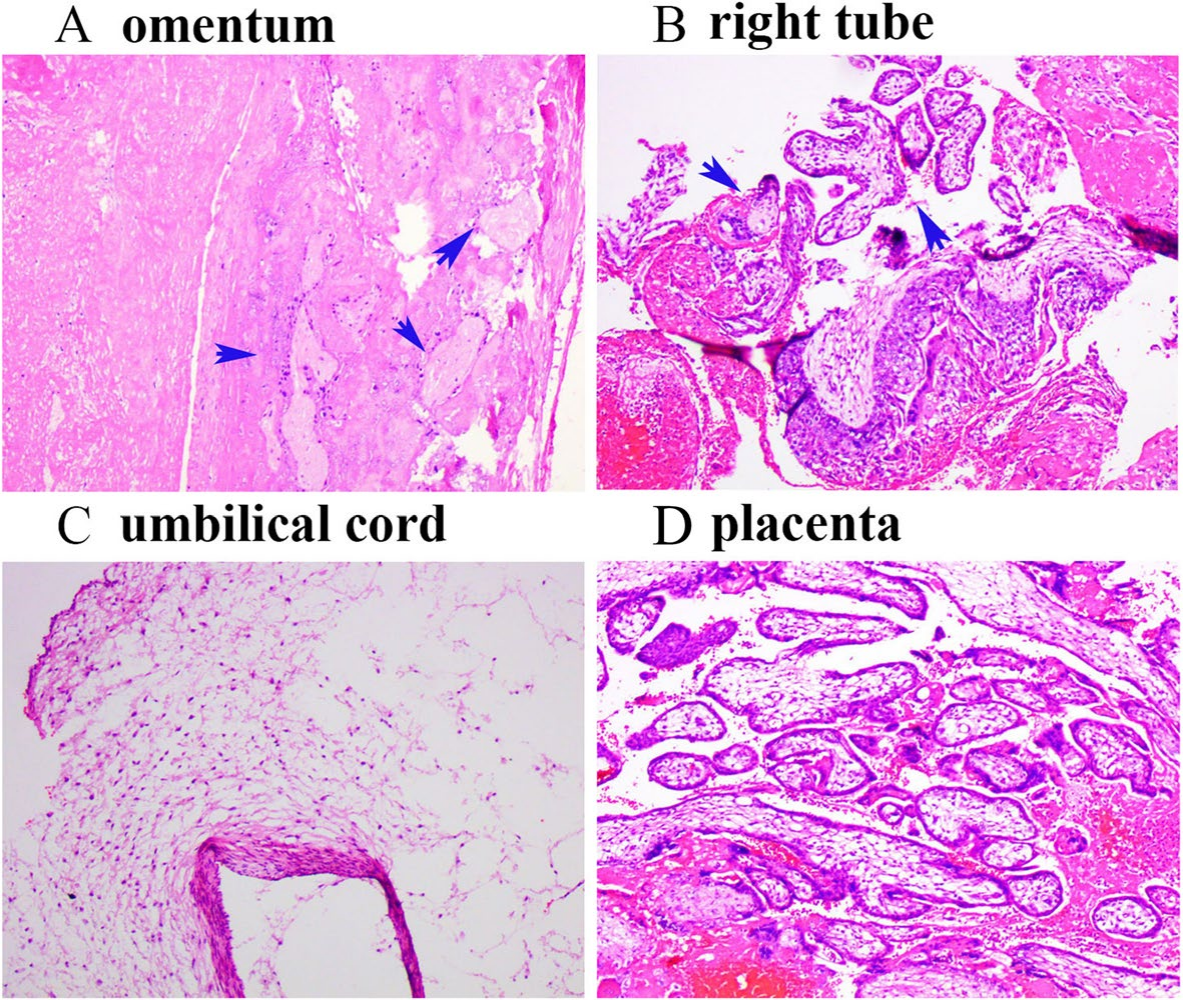
Postoperative pathological images. Blue arrows indicate the chorionic villi

Postoperative pathological results (Fig. 3) confirmed the secondary abdominal pregnancy because decidua tissue and chorionic villi both implanted into the right tube and omentum. Seven days after the operation, she was discharged. Two weeks later, during an outpatient follow-up visit, the patient was doing well, without complaints. Till now, one year past, she was married again but has weak fertility awareness because of her catastrophic experiences, and she was experiencing anxiety, depression after pregnancy loss.

## Discussion and conclusions

### Pathogenesis and definition

Pathogenesis of APs is still controversial. Primary and secondary ones are classified. Pathogenesis mostly identified intraoperatively, confirmed pathologically. Primary peritoneal implantation is rare, and Studdiford proposed criteria for its diagnosis include the following:(1) normal tubes and ovaries, (2) absence of uteroplacental fistula, and (3) sufficiently early diagnosis to exclude the possibility of secondary implantation. The majority of cases are usually secondary implantation. Using the site of placental implantation as the key to distinguish pathogenesis of the secondary APs, Clark JF revealed that APs secondary to tubal abortion, tubal rupture and rupture or perforation of the uterus [18]. Most of advanced APs are usually secondary resulting from aborted or ruptured tubal pregnancy [19].

The definition of an AP is still obscure and controversial. AP has historically been defined as an ectopic pregnancy implanting in the peritoneal cavity [4]. It is obscure and less effective in secondary APs, especially late ones beyond the first-trimester. The definition of a late APs has been debated over the association between the placenta and the primary implant site [20, 21]. In 2008, Kevin C. Worley, MD etc. proposed that in advanced extrauterine pregnancy, irrespective sites of placenta, a more clinically useful definition of abdominal pregnancy is an extrauterine pregnancy in which all or most of the fetus develops within the abdominal [21]. But Mahajan NN argued that implantation of the placenta in the peritoneal cavity by Atrash et al. is a more logical and practical definition [20].

According the sites of placenta insertion in our case it can be classified as tubo-abdominal pregnancy, an uncommon form of APs secondary to ruptured tubal pregnancy. At the time of operation in this case, a particular phase of pathogenesis of secondary AP developed, secondary implantation of partial placenta on momentum occurred synchronously partial placenta remained in the primary implanted tube, with a living fetus abdominal growth. Removal of both implantation sites had been done, and the histopathology identified the insertion of villi in both the oviduct and the omentum specimens. Tubo-abdominal pregnancy, which was coined ever in 1910 by McCann F [22], reported by Clark JF in 1966 [18] and by E Holzer in 1976 [23], but rarely mentioned in current cases of APs, it may be explained by the rarity itself and the changes of occurrence of EPs. The delayed presentation allowed for the dynamic pathogenesis of the secondary AP, or the unique form of secondary APs to be clarified once again, tubo-abdominal pregnancy was firstly explicitly classified as secondary APs by Clark JF in his table. This case serves as a timely reminder to clinicians that patients’education is crucial to make early diagnosis.

### History and presentation

The clinical symptom of abdominal pregnancy is variable and nonspecific, depending on the degree of the anatomical distortion it creates and the placental insertion site. But the following signs should bring the practitioner to think about the diagnosis: abdominal pain, bloody vaginal discharge, gastrointestinal symptoms, altered bowel movements, even painful fetal movements in APs [6, 24]. Risk factors for APs are low socioeconomic status, pelvic inflammatory disease, history of infertility, intrauterine device, endometriosis and assisted reproductive techniques. IVF is a known risk of APs in several trends including tubal factor infertility, history of tubal ectopic and tubal surgery, higher number of embryos transferred, and fresh embryo transfers and so on, many strategies are employed to reduce the incidence of APs in fertility treatment [25–27].

In our case, she conceived naturally this time with her boyfriend. Except for infertility as the risk factor, previous IVF history should not be neglected. Puncture of ovarian in previous IVF procedure maybe to some extent account for the inflammation confirmed on both ovaries intraoperatively. The most importantly, women with IVF history may be high risk population to experience intended pregnancies. Fixed belief in infertility throughout her previous IVF treatment was a major barrier to contraception use resulting in an unexpected pregnancy [28], which raised many issues. The lack of information on the probability of spontaneous pregnancies (intrauterine or extrauterine ones) during and after the IVF procedure [28, 29], so she was unlikely to engage with counselling in department of Obstetrics and gynecology when symptoms existed. A missed and delayed AP still occurred in modern medicine. The views expressed by the women in our report is widely held by most infertile couples having IVF history. Our case sampled that fertility providers, could and should do more on educational interventions, healthcare professionals must all be armed with knowledge specific to women having had IVF history.

Moreover, eutopic or ectopic pregnancy is a competition between signals derived from the endometrium and the fallopian tube for blastocyst implantation, it is a complicated hormonally regulated process [30, 31]. As a steroid hormone discorded disease, PCOS was associated with an increased risk of EP after controlled ovarian hyperstimulation in fresh embryo transplantation cycles [32], it may be an unknown risk factor need to be focused on in spontaneous EP research in future. RIF history in this PCOS woman who got abdominal pregnancy, partially indicate endometrial receptivity maybe a reason need pay attention to [33]. At last, in intra-uterine insemination cycles, EP is associated with sperm source [34], although she got APs with her new partner, but little evidence is available to answer the male factor involved in the etiology of this spontaneous AP.

### Diagnosis and management

Ultrasonography is available, non-invasive method which can allow to distinguish the APs. The sonographic criteria proposed by Gerli et al.in 2004 can be followed in the first-trimester to make a diagnosis of APs [35]. The APs diagnosed in early gestation is usually confirmed as primary ones by laparoscopy and managed successfully. In the second trimester, APs can be diagnosed using the following criteria proposed by Allibone et al. in 1982:Empty uterine cavity; No evidence of a dilated Fallopian tube or complex adnexal mass; Gestation sac surrounded by loops of bowel and separated by peritoneum; Wide mobility similar to fluctuation of the sac [36]. But the most frequent and reliable finding was separation of the uterus from the fetus (90%) and extrauterine placenta (75%) [37]. In the late individual cases, especially when a rupture occurs, they still present a diagnostic dilemma. In our case, a second-trimester AP was diagnosed preoperatively, but accurate detection of site of placental implantation and relationship with surrounding tissue was challenging in an emergent situation by sonography. Magnetic resonance imaging (MRI) which is better for clarifying anatomic relationship with surrounding structures, vascular supply, placental site, and unusual fetal lie [38], but it is not available in our patient who presented with shock requiring immediate surgical intervention for life saving. A laparotomy approach was chosen individually. As revealed intraoperatively, the rupture occurred at primary implanted site rather than secondary implanted site, the placenta partially inserted into the omentum, notably, it was the early stage of pathogenesis of secondary APs and the lack of broadly and deeply trophoblastic invasion into other important organs alleviated the difficulty of placental dissection and removal of the conception products.

### Conclusion

It was unique to report an unexpected spontaneously APs in PCOS woman with a previous IVF history. “Self-identified” infertility led to a missed and delayed second-trimester APs, it was a case need laparotomy in emergency, with a loss of opportunity for medical treatment, and severe damage to the woman’s subsequent reproductive potential. Diagnosis and management modalities in EPs and APs should be a multidisciplinary systematic team work that includes high-risk population identification, provision of pregnancy and contraception knowledge, early diagnosis, appropriate management and available strategies for re-pregnancy and re-contraception after EPs and APs. A tubo-abdominal pregnancy mentioned again, serving as a timely reminder to clinicians, with the widely availability of artificial reproductive technology in maternal and child health care, focusing on educational interventions in women having previous IVF history is primitive prevention of EPs and APs, with an important socioeconomic value.

## Supplementary Information

**Additional file 1.**

## Data Availability

The datasets created during and/or analyzed during the current study available from the corresponding author on reasonable request.

## Abbreviations

EPs: Ectopic pregnancies
Aps: Abdominal pregnancies
hCG: Human chorionic gonadotropin
PCOS: Polycystic ovarian syndrome
IVF: In vitro fertilization
RIF: Repeated implantation failure (RIF)
